# Biological and Physico-Chemical Properties of Composite Layers Based on Magnesium-Doped Hydroxyapatite in Chitosan Matrix

**DOI:** 10.3390/mi13101574

**Published:** 2022-09-22

**Authors:** Simona Liliana Iconaru, Carmen Steluta Ciobanu, Gabriel Predoi, Krzysztof Rokosz, Mariana Carmen Chifiriuc, Coralia Bleotu, George Stanciu, Radu Hristu, Steinar Raaen, Stefania Mariana Raita, Liliana Ghegoiu, Monica Luminita Badea, Daniela Predoi

**Affiliations:** 1 National Institute of Materials Physics, 405A Atomistilor Street, 077125 Magurele, Romania; 2 Faculty of Veterinary Medicine, University of Agronomic Sciences and Veterinary Medicine of Bucharest, 105 Splaiul Independentei, 050097 Bucharest, Romania; 3 Faculty of Electronics and Computer Science, Koszalin University of Technology, Sniadeckich 2, PL 75-453 Koszalin, Poland; 4 Life, Environmental and Earth Sciences Division, Research Institute of the University of Bucharest (ICUB), University of Bucharest, 060023 Bucharest, Romania; 5 Academy of Romanian Scientists, 54 Splaiul Independentei Street, 050085 Bucharest, Romania; 6 Biological Sciences Division, The Romanian Academy, 25 Calea Victoriei, 010071 Bucharest, Romania; 7 Stefan Nicolau Virology Institute, 285 Mihai Bravu Avenue, 030304 Bucharest, Romania; 8 Center for Microscopy-Microanalysis and Information Processing, University Politehnica of Bucharest, 313 Splaiul Independentei, 060042 Bucharest, Romania; 9 Department of Physics, Norwegian University of Science and Technology (NTNU), Realfagbygget E3-124 Høgskoleringen 5, NO 7491 Trondheim, Norway; 10 Faculty of Horticulture, University of Agronomic Sciences and Veterinary Medicine, 59 Marasti Boulevard, 011464 Bucharest, Romania

**Keywords:** magnesium, hydroxyapatite, morphology, biocompatibility, antimicrobial properties

## Abstract

In the present study, we report the development and characterization of composite layers (by spin coating) based on magnesium-doped hydroxyapatite in a chitosan matrix, (Ca_10−x_Mg_x_(PO_4_)_6_(OH)_2_; x_Mg_ = 0, 0.08 and 0.3; HApCh, 8MgHApCh and 30MgHApCh). The MgHApCh composite layers were investigated using scanning electron microscopy (SEM), energy-dispersive X-ray spectroscopy (EDX), and X-ray photoelectron spectroscopy (XPS) techniques. The in vitro biological evaluation included the assessment of their cytotoxicity on MG63 osteoblast-like cells and antifungal activity against *Candida albicans* ATCC 10231 fungal cell lines. The results of the physico-chemical characterization highlighted the obtaining of uniform and homogeneous composite layers. In addition, the biological assays demonstrated that the increase in the magnesium concentration in the samples enhanced the antifungal effect but also decreased their cytocompatibility. However, for certain optimal magnesium ion concentrations, the composite layers presented both excellent biocompatibility and antifungal properties, suggesting their promising potential for biomedical applications in both implantology and dentistry.

## 1. Introduction

Today, the development of new biomaterials, including coatings, with enhanced biological properties are of real interest for the scientific world due to the exponential growth of demand for such materials [1]. In this context, composite coatings based on hydroxyapatite (HAp) have seen rapid development in recent years due to their good biocompatibility, bioactivity and osteoconductivity, which make them sui for therapeutic uses in implantology and dentistry [2,3]. Previous studies regarding the biological and physico-chemical properties of doped HAp [4,5,6,7] have shown that by doping hydroxyapatite with various ions, its properties are enhanced [4,5,6,7], making it appropriate for the development of new biomaterials with potential applications in the biomedical field.

From these ions, magnesium (Mg^2+^) is a chemical element with exceptional biological properties, being the prevalent divalent cation that can be found in the intracellular compartment and also the fourth most abundant cation in the body, playing a very important role in various biological processes such as the synthesis of nucleic acids [8]. The human body has about 24 g of Mg^2+^, and bone tissue contains approximately 50–60% of it, leaving most of the rest stored in soft tissues and less than 1% of the total body Mg^2+^ in the blood [9,10,11,12,13,14,15,16]. Furthermore, studies conducted on bodily Mg^2+^ kinetics have shown that intracellular magnesium concentrations have an interval between 5 to 20 mmol/L [13,14,17]. From this, from 1% to 5% can be found ionized, while the rest can be found bound to proteins, or negatively charged molecules and adenosine triphosphate (ATP) [17].

Due to its outstanding biocompatibility, HAp promotes the regeneration of various types of soft tissues [18]. The biocompatibility of HAp and doped HAp was studied using various cell lines including human osteoblast-like cells (MG63) [19,20]. The results of the study, conducted by Begam et al. [10] on pure and zinc-doped hydroxyapatite, revealed that enhanced biological properties were obtained for the doped samples. Overall, the best MG63 cell response was obtained for the HAp samples sintered at 1250 °C [19]. In addition, Geng and collaborators [21] reported that MG63 cells adhere and proliferate easier on the surface of β-tricalcium phosphate coatings deposited on Mg substrates than on uncoated Mg substrates. Moreover, they reported a cell viability under 80% for pure Mg substrates, which indicates the cellular toxicity of this substrate [21].

The use of biomaterials in biomedical applications that do not possess antimicrobial properties comes with an increased risk of developing an infection associated with the implant [8,22]. One of the most common fungal species involved in the formation of biofilm and the occurrence of post-implant fungal infections is *Candida albicans* (*C. albicans*) [23,24]. In our previous study, we reported that magnesium-doped hydroxyapatite (Ca_10−x_Mg_x_(PO_4_)_6_(OH)_2_, x_Mg_ = 0.1) has an effective antimicrobial activity against *C. albicans* [25].

Chitosan (Ch), described with the chemical formula C_56_H_103_N_9_O_39,_ is a naturally occurring linear polysaccharide mainly composed from glucosamine, (C_6_H_13_NO_5_), having a wide range of applications in the biomedical domain [26,27,28,29,30]. Therefore, the fabrication of new composite coatings based on magnesium-doped hydroxyapatite in a chitosan matrix (with various Mg concentrations) are of great interest due to their potential properties and applications in the medical field.

Previous studies conducted on hydroxyapatite/chitosan and doped hydroxyapatite/chitosan composite coatings highlighted the fact that the biological properties of these biomaterials are influenced by the deposition conditions and by their exposure to various doses of gamma radiation [31,32,33].

Consequently, the aim of this paper was to develop and to evaluate the biological and physico-chemical properties of magnesium-doped hydroxyapatite in a chitosan matrix (Ca_10__−x_Mg_x_(PO_4_)_6_(OH)_2_; x_Mg_ = 0, 0.08 and 0.3; HApCh, 8MgHApCh and 30MgHApCh)composite coatings. Secondly, the influence of the magnesium concentration on the biological and physico-chemical properties was also studied. For this purpose, the chemical composition of the coatings obtained by spin coating was evaluated by energy-dispersive X-ray spectroscopy (EDX), and X-ray photoelectron spectroscopy (XPS). The surface morphology was also analyzed by scanning electron microscopy (SEM).

The cytocompatibility of the HApCh, 8MgHApCh and 30MgHApCh coatings was evaluated with the aid of Human osteoblast-like cell lines (MG63), and their antimicrobial activity was tested against a *C. albicans* ATCC 10231 fungal strain. Our results suggested the potential of HApCh, 8MgHApCh and 30MgHApCh composite coatings for developing new biomaterials with applications in implantology and dentistry.

## 2. Materials and Methods

### 2.1. Materials

The reagents that were used for the fabrication of magnesium-doped hydroxyapatite in a chitosan matrix (Ca_10__−x_Mg_x_(PO_4_)_6_(OH)_2_; x_Mg_ = 0, 0.08 and 0.3) by the sol-gel method were: calcium nitrate tetrahydrate (≥99.0%, Sigma Aldrich, St. Louis, MO, USA), diammonium hydrogen phosphate (≥99.0%, Sigma Aldrich, St. Louis, MO, USA) and magnesium nitrate hexahydrate (99.97%; Alpha Aesar, Kandel, Germany). In addition, other chemicals used in the fabrication process, such as chitosan, ethanol absolute and ammonium hydroxide, were procured from Sigma Aldrich (St. Louis, MO, USA). Si wafer (the substrate) was purchased from Siegert Wafer GmbH (Aachen, Germany).

### 2.2. Preparation of Magnesium-Doped Hydroxyapatite in a Chitosan Matrix

The synthesis of magnesium-doped hydroxyapatite in a chitosan matrix (Ca_10__−x_Mg_x_(PO_4_)_6_(OH)_2_; x_Mg_ = 0, 0.08 and 0.3) was conducted following our previous studies [25,31]. For this purpose, the value used for the Ca/P molar ratio was 1.67. Therefore, the magnesium-doped hydroxyapatite was obtained by an adapted method in ambient conditions. The calcium nitrate tetrahydrate and magnesium nitrate hexahydrate were dissolved together and afterwards added in a diammonium hydrogen phosphate solution [25]. During the synthesis, the solution pH was maintained at 11 with the aid of ammonium hydroxide solution. The final suspension was stirred at 80 °C and then centrifuged and washed several times [25]. The final suspension was added in a 2% chitosan solution and stirred for 4 h at 100 °C on a hot plate, in ambient conditions. Finally, the obtained gel suspensions were: HApCh (Ca_10__−x_Mg_x_(PO_4_)_6_(OH)_2_; x_Mg_ = 0); 8MgHApCh (Ca_10__−x_Mg_x_(PO_4_)_6_(OH)_2_; x_Mg_ = 0.08) and 30MgHApCh (Ca_10__−x_Mg_x_(PO_4_)_6_(OH)_2_; x_Mg_ = 0.3). All these gel suspensions were used in order to develop the HApCh and MgHApCh coatings.

### 2.3. Fabrication of HApCh and MgHApCh Coatings

The HApCh and MgHApCh coatings were obtained by spin-coating in agreement with our previous paper [18]. The coatings were obtained by dripping 0.5 mL of the obtained gel suspension on the Si wafer under the following conditions: the speed was set at 2000 rpm and the spin time used was equal to 90 s. This step was conducted 3 times consecutively. Finally, the obtained coatings were thermally treated in air at 500 °C for 2 h. This process was performed in order to obtain each type of coating, namely HApCh (Ca_10__−x_Mg_x_(PO_4_)_6_(OH)_2_; x_Mg_ = 0); 8MgHApCh (Ca_10__−x_Mg_x_(PO_4_)_6_(OH)_2_; x_Mg_ = 0.08) and 30MgHApCh (Ca_10__−x_Mg_x_(PO_4_)_6_(OH)_2_; x_Mg_ = 0.3).

### 2.4. Physico-Chemical Studies

The XPS observations were realized with the aid of a SES 2002 instrument (Scienta Omicron, Taunusstein, Germany). The instrument used a monochromatic Al K(alpha) (hν = 1486.6 eV) X-ray source (Scienta Omicron, 18.7 mA, 13.02 kV, Taunusstein, Germany). For the analysis of the acquired XPS data, the Casa XPS 2.3.14 software (Shirley background type, Casa Software Ltd, Las Vegas, NV, USA) was used [34]. All the values obtained for the binding energy (BE) reported in this paper were charge-corrected to C 1s at 284.8 eV. The functional groups and composition of the obtained coatings were identified by Fourier transform infrared spectroscopy (FTIR) using a Perkin Elmer SP-100 spectrometer (Waltham, MA, USA). The FTIR equipment was operated in the ATR (Attenuated Total Reflection) mode. The ATR-FTIR spectra were acquired in the wavenumber range between 400 and 3900 cm^−1^.

The coating morphology and chemical compositions were observed by scanning electron microscopy (SEM) using a Hitachi S4500 microscope (Hitachi, Tokyo, Japan) equipped with an energy-dispersive X-ray spectroscopy (EDX) device. The SEM micrographs were acquired at 50,000× magnification.

### 2.5. Biological Evaluation

#### 2.5.1. Fluorescein Diacetate (FDA)—Propidium Iodide (PI) Cell Staining

MG63 (10^5^ cells) were seeded in 24-well plates containing coated and uncoated materials. The cells were maintained for 48 h in Dulbecco’s Modified Eagle Medium (DMEM); F12 supplemented with 10% fetal bovine serum. Then, the cells were stained with a solution containing 100 µg/mL fluorescein diacetate (FDA) and 50 µg propidium iodide (PI). After 5 min of incubation in the dark, the cells were examined with an Observer D1 Zeiss microscope (Carl Zeiss Microscopy GmbH, Jena, Germany) using a bandpass 488 nm exciter filter.

Supplementary information about the MG63 cell attachment and proliferation on the surface of studied composite coatings were provided by atomic force microscopy (AFM) studies. Therefore, for this aim, the cells were fixed using 70% ethanol before AFM investigation. After this step, the AFM images of the coating surface were recorded on a surface area of 50 µm × 50 µm in ambient conditions with the help of an NT-MDT NTEGRA Probe Nano Laboratory instrument (NT-MDT, Moscow, Russia). The obtained AFM images were analyzed using the Gwyddion 2.59 software (Department of Nanometrology, Czech Metrology Institute, Brno, Czech Republic) [35].

In addition, data regarding the cell attachment and proliferation on the composite coatings were obtained with the aid of an inversed trinocular metallographic microscope (MM) OX.2153-PLM (Euromex, Arnhem, The Netherlands). The MM images were obtained using an objective with a 20× magnification.

#### 2.5.2. Analysis of Cell Cycle

The MG63 cells (10^5^ cells) were maintained for 48 h in the presence of coated and uncoated materials, trypsinized, washed in cold phosphate buffered saline (PBS) and then fixed in 70% ethanol for at least half an hour at −20 °C. The cells were washed with PBS, treated with RNase A (1 mg/mL) and stained with propidium iodide (PI) (100 μg/mL) at 37 °C for 1 h. Cellular DNA content was measured using Beckman Coulter EPICS XL flow cytometer (Beckman Coulter, Brea, CA, USA) and analyzed with the FlowJo 8.8.6 software program (Ashland, OR, USA).

#### 2.5.3. In Vitro Antifungal Assays

The antifungal properties of the MgHApCh composite layers were investigated by in vitro assay against the reference *Candida albicans* ATCC 10231 fungal strain. The antifungal studies were performed according to [33] and the antifungal efficiency of the composite layers was determined for three different time intervals of incubation (24, 48 and 72 h). The experiments were carried out in triplicate and the results were depicted as mean ± standard deviation (SD). The qualitative evaluation of the fungal cells’ adherence and proliferation on the surface of the composite layers was carried out with the aid of confocal laser scanning microscopy (CLSM) studies. For this purpose, *C. albicans* fungal cells were grown on the surface of both Si discs and MgHApCh composite layers. Afterwards, the Si discs and the composite thin films were removed from the culture medium, washed with sterile saline solution for the removal of unattached fungal cells and fixed with cold methanol. Before the CLSM visualization, the samples were stained in the dark using ethidium bromide and visualized in reflection and fluorescence modes. The CLSM visualization was performed with a Leica TCS-SP confocal microscope (Wetzlar, Germany) equipped with a PL FLUOTAR (40_NA 0.7) objective and operated in reflection and fluorescence modes and an Ar ion laser (488 nm) was also used [36]. Details about the morphology of the *C. albicans* fungal cells were attained using ImageJ software (Image J 1.51j8, National Institutes of Health, Bethesda, MD, USA [37]).

## 3. Results

### 3.1. Structural and Morphological Characterizations

**Figure 1:**
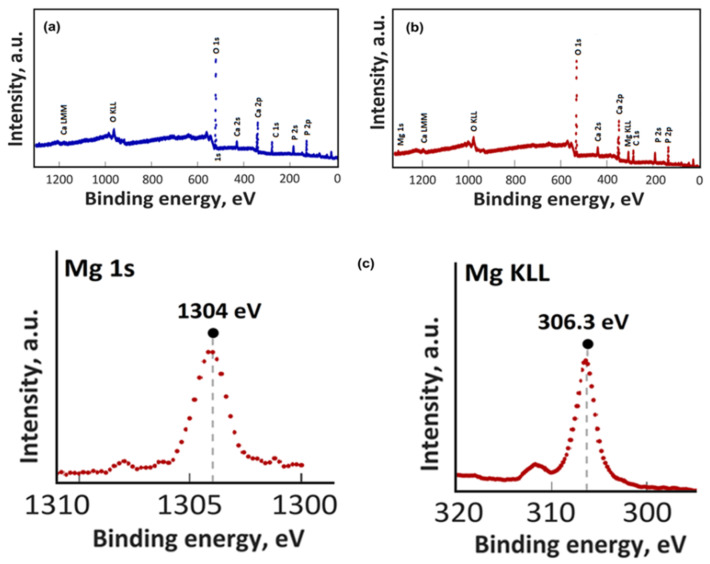
XPS survey results of HApCh (**a**), 30MgHApCh (**b**) and XPS high resolution spectra of Mg 1s and Mg KLL of 30MgHApCh (**c**).

The XPS evaluation of HApCh, 8MgHApCh and 30MgHApCh layers was obtained. The presence of magnesium ions in the 8MgHApCh and 30MgHApCh layers was shown. Figure XPS revealed the general XPS spectra of the HApCh and 30MgHApCh layers. The constituent elements such as Ca, P, O, Mg and C were identified (Figure 1a,b).

In Figure 1c, the XPS high resolution spectra of Mg 1s and Mg KLL of the 30MgHApCh layers are shown. The peak position of Mg 1s for the 30MgHApCh layers (Figure 1c) was detected at binding energies (BE) of 1304 eV and the peak position of Mg KLL was located at a BE of 306.3 eV.

**Figure 2:**
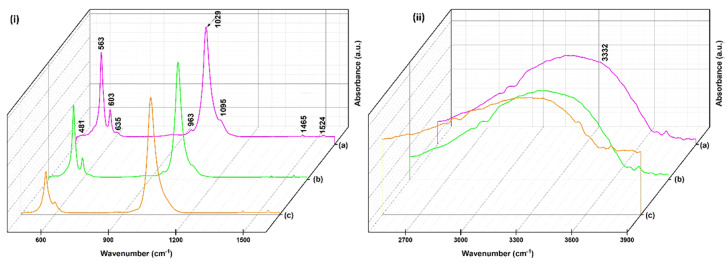
Three-dimensional representation of absorbance FTIR spectra obtained on HapCh (a), 8MgHApCh (b) and 30MgHApCh (c) composite coatings. The FTIR spectra are presented in 400–1600 cm^−1^ (**i**) and 2500–3900 cm^−1^ (**ii**) spectral range.

The FTIR spectra of the magnesium-doped hydroxyapatite in a chitosan matrix with various Mg^2+^ concentrations (HApCh, 8MgHApCh and 30MgHApCh) are shown in Figure 2. In the ATR-FTIR spectra of the HApCh composite coatings, the intense bands observed at 1095, 1029 and 963 cm^−1^ belonged to the stretching modes of PO_4_^3−^ [32,33]. Furthermore, other intense adsorption peaks were noticed at 481, 563, 603 and 635 cm^−1^, these being characteristic to the bending modes of phosphate groups (ν_2_ and ν_4_) from the hydroxyapatite structure [32,33]. Weak adsorption bands observed at 1465 and 1524 cm^−1^ were specific to the chitosan structure [32,33]. Namely, the adsorption peak found at 1524 cm^−1^ was attributed to the NH_2_ groups from chitosan [32,33]. Furthermore, the adsorption bands of the –NH_2_ group from the chitosan structure noticed at 1465 cm^−1^ were overlapped with the adsorption bands of the carbonate groups from the HAp [38,39,40]. In addition, the broad band from 3332 cm^−1^ (specific to hydroxyl groups from HAp structure), was overlapped with the adsorption bands of the –NH_2_ group [38,39,40].

In the case of the 8MgHApCh and 30MgHApCh coatings, it was observed that the adsorption bands were slightly displaced compared to the ones of HApCh coatings. Overall, a decrease in the peak intensity was observed with the increase in magnesium concentration in the samples. This behavior could indicate a decrease in the coating’s crystallinity. Therefore, all the characteristic adsorption peaks of hydroxyapatite and chitosan were present in the FTIR spectra, suggesting the formation of magnesium-doped hydroxyapatite in a chitosan matrix [32,33]. The IR wavenumber (cm^−1^) assignments of the HApCh sample are summarized in Table 1.

**Figure 3:**
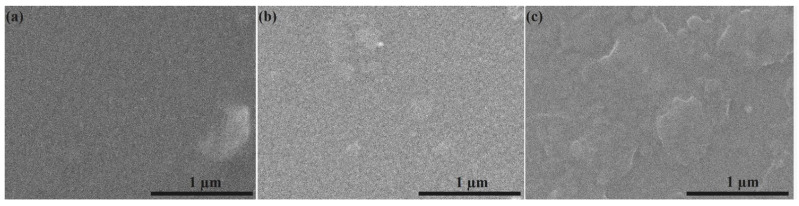
SEM micrographs obtained for HApCh (**a**), 8MgHApCh (**b**) and 30MgHApCh (**c**) composite coatings.

The SEM micrographs obtained for the HApCh (Figure 3a), 8MgHApCh (Figure 3b) and 30MgHApCh (Figure 3c) coatings are presented in Figure 3. The SEM images highlighted the smooth surfaces of the studied coatings. On the other hand, it was noticed that, for the sample with the highest Mg concentration (30MgHApCh), the surface was more structured compared with the surface of the HApCh coatings. Furthermore, on the surface of the coatings, the presence of surface defects, such as cracks, was not observed.

**Figure 4:**
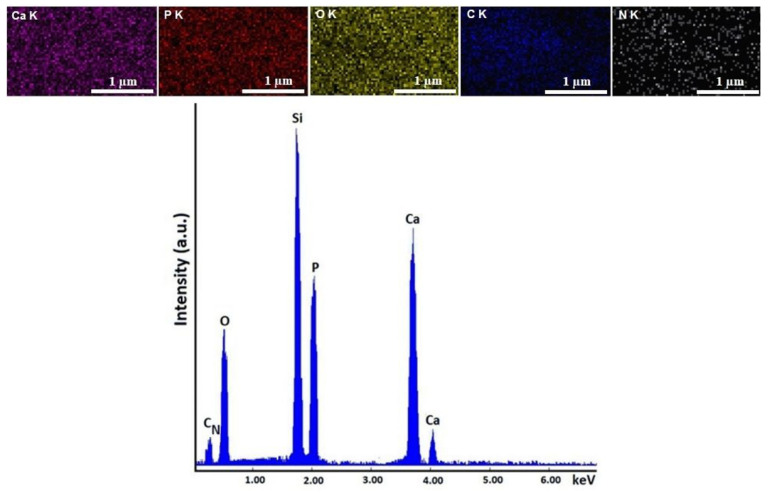
Elemental mapping distribution and EDX spectra of HApCh composite coatings.

**Figure 5:**
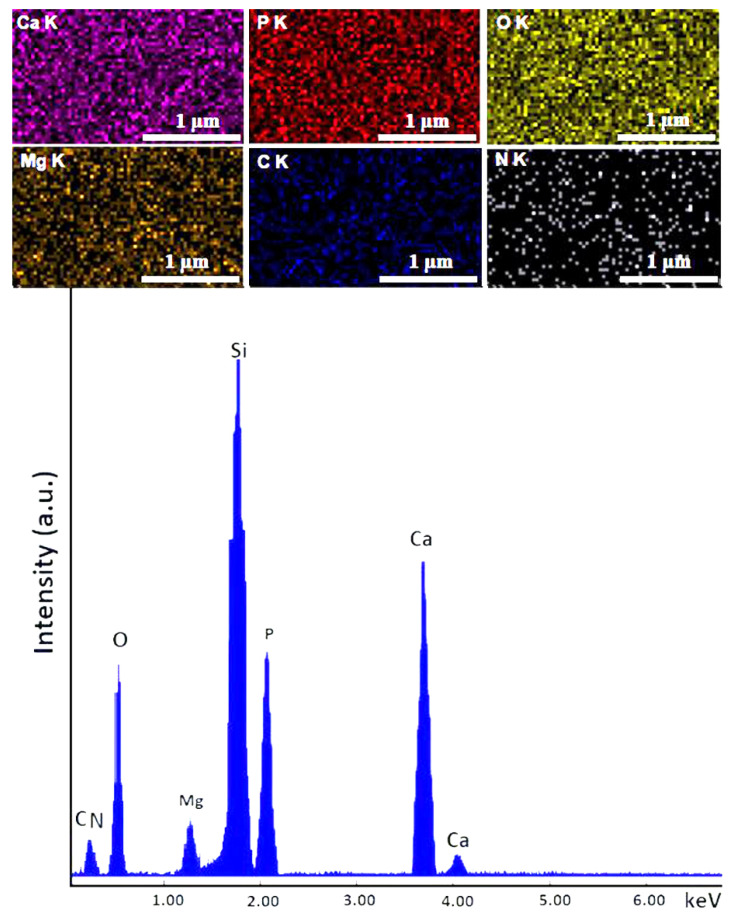
Elemental mapping distribution and EDX spectra of 30MgHApCh composite coatings.

EDX studies were used in order to investigate the chemical composition of the obtained coatings. Therefore, the results of the EDX studies along with the elemental distribution maps of the main elements are presented in Figure 4 and Figure 5.

The EDX spectra obtained for the HApCh composite coatings (Figure 4) show the presence of calcium, phosphorus, oxygen, carbon and nitrogen in the coatings. All these chemical elements belonged to the HAp and chitosan. In addition, the homogenous distribution of these chemical elements throughout the composite coatings was sustained by the elemental distribution maps presented in the Figure 4.

Similar results were also obtained for the 30MgHApCh composite coatings (Figure 5). In the EDX spectrum, besides the presence of calcium, phosphorus, oxygen, carbon and nitrogen, the presence of magnesium was also observed. In all the EDX spectra, the presence of an Si line (which was due to the substrate) was observed. No other lines were noticed in the obtained EDX spectra, which highlighted the purity of the analyzed layers.

The concentration of Ca, P, O and Mg (at %) obtained from the EDX studies conducted on the composite coatings are revealed in Table 2.

### 3.2. Biological Evaluations

**Figure 6:**
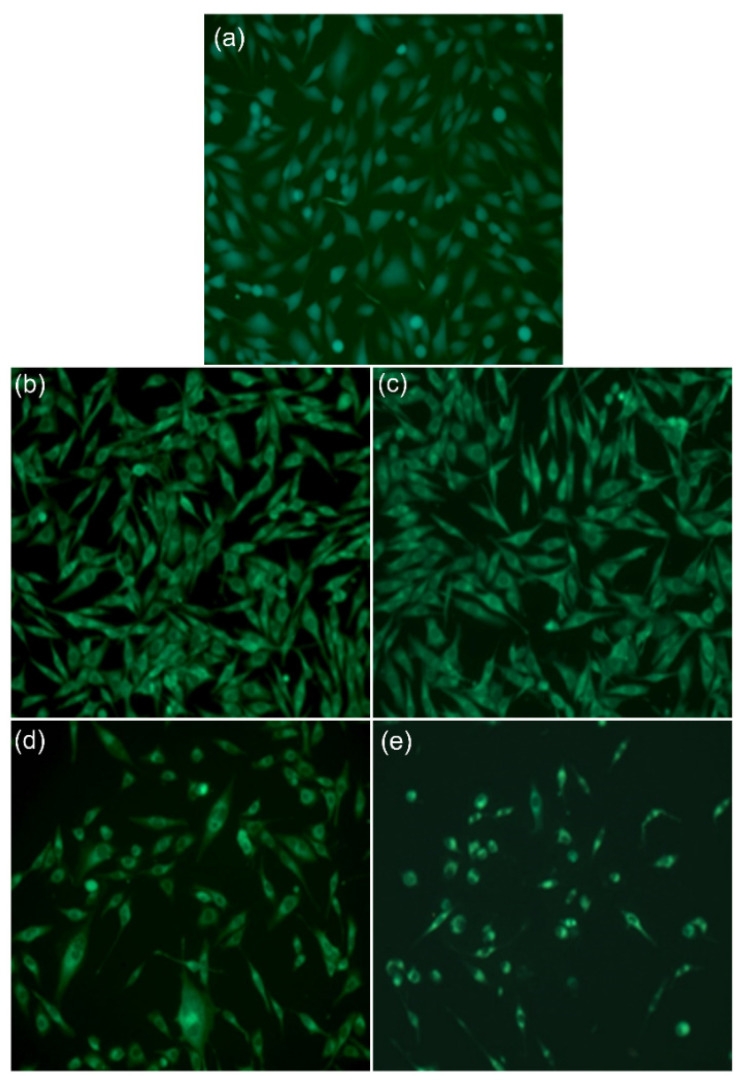
The morphology of MG63 cells grown on coated materials: control (**a**), Si (**b**), HApCh (**c**), 8MgHApCh (**d**), 30MgHApCh (**e**) (Fluorescence, Objective 20×).

The coated materials were placed in 24-well plates for toxicity evaluation, and MG63 cells were added at a concentration of 10^5^ cells/well. We allowed the cells to grow on these materials for 48 h and then stained them with a combination of the following dyes: FDA (staining the viable cells) and PI (staining the dead cells). The cells grown on the coated material retained the FDA dye, thus being viable, but a decrease in the viable cells number was observed in the presence of Mg, with the very rare appearance of dead cells (stained red). We noticed that, in the presence of Mg, the morphology of the human osteoblast-like cells (MG63) was modified: the cells were more rounded, the cytoplasm was reduced, and the nucleus had condensed chromatin (Figure 6).

**Figure 7:**
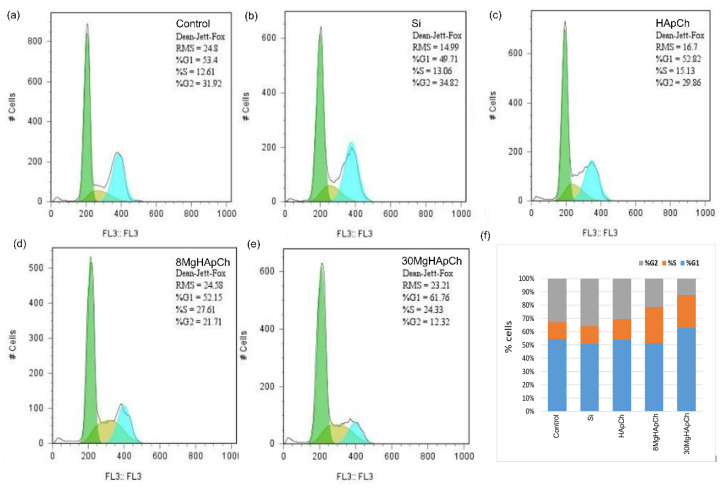
Flow cytometry analysis of MG63 cells grown in the presence of different substrates. Control (**a**), Si (**b**), HApCh (**c**), 8MgHApCh (**d**), 30MgHApCh (**e**) (G1-green, S-yellow, G2-blue) and graphical representation of MG63 cell cycle parameters (**f**).

Flow cytometry analysis of cells grown in the presence of the MgHApCh composite layers showed that, in the presence of Mg, there was a slight increase in the number of cells found in the synthesis (S) phase and a more significant reduction in the number of cells in G2/M in the case of the highest Mg concentration (x_Mg_ = 0.3) (Figure 7). All these results supported the toxicity induced by the presence of Mg.

**Figure 8:**
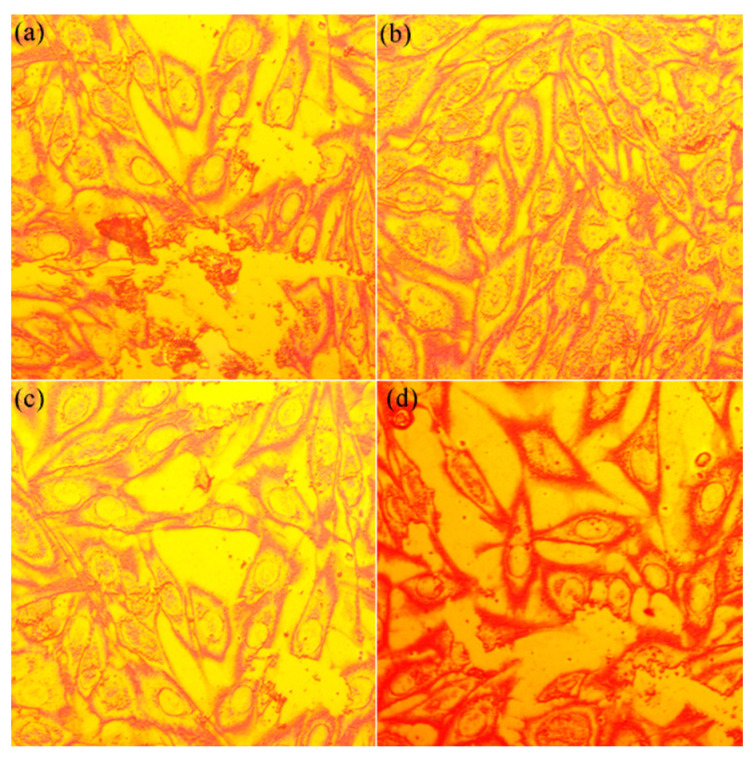
Metallographic microscopy images of MG63 cells after 48 h of incubation with Si discs (**a**), HApCh (**b**), 8MgHApCh (**c**) and 30MgHApCh (**d**) composite layers.

Additional information regarding the biocompatibility of the MgHApCh composite layers after 48 h of incubation with MG63 human-like osteoblast cells was obtained using metallographic microscopy (MM). The results of the MM visualization are presented in Figure 8a–d.

The images obtained with the metallographic microscopy suggested that, after 48 h of incubation with the Si discs and MhHApCh composite layers, the MG63 cells preserved their morphology almost entirely in the presence of the HApCh and 8MgHApCh composite layers, but modification occurred for the cells incubated with the Si discs and 30MgHApCh composite layers. The occurrence of rounded cells was noticed even in the case of the 8MgHApCh (Figure 8c) composite layers, but a more pronounced effect on the MG63 cells’ morphology was observed in the case of Si discs (Figure 8a) and 30MgHApCh composites layers (Figure 8d). These results agree with the results obtained from the optical microscopy and they strongly highlighted the fact that the MgHApCh composite layers were favorable to MG63 cell adherence and development. Studies have demonstrated that elongated cells present a higher adhesion behavior than spherically shaped cells. This behavior could be explained by the higher density of adhesion focal points of elongated cells and their better organization of the cytoskeleton, supported by stronger actin fibers [41,42,43,44,45].

**Figure 9:**
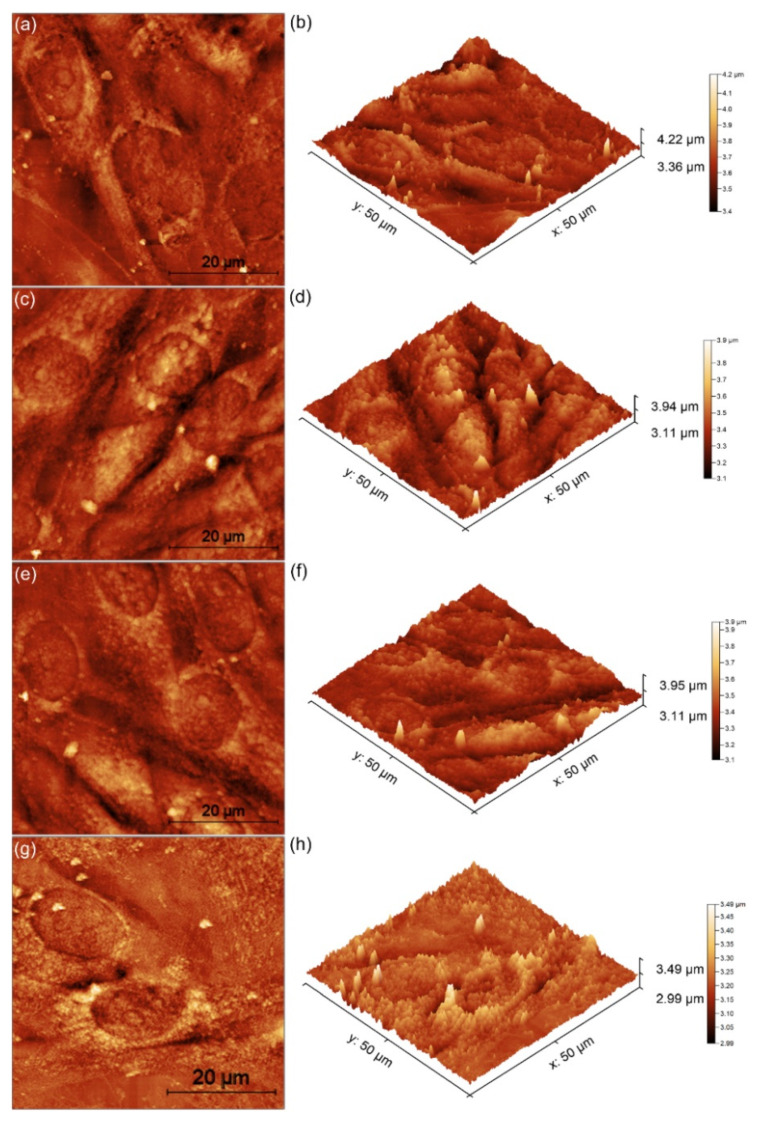
Two-dimensional AFM topography of MG63 cells after 48 h of incubation with Si discs (**a**), HApCh (**c**), 8MgHApCh (**e**) and 30MgHApCh (**g**) composite layers and their 3D representation (**b**,**d**,**f**,**h**).

The adhesion and morphology of the MG63 cells incubated for 48 h on the surface of the Si discs and MgHApCh composite layers were also examined by AFM. The AFM topographies were recorded on surfaces of 50 × 50 µm^2^ in normal atmospheric conditions and at room temperature. The 2D topographies and the respective 3D representations of the MG63 cells adhered on the surfaces of the Si discs and MgHApCh composite layers after 48 h of incubation are presented in Figure 9a–h.

The AFM results showed that the MG63 cells adhered to all the tested surfaces. More than that, the results suggested that, for the HApCh samples and 8MgHApCh samples, there were not any major changes observed in the typical morphology of the MG63 cells. On the other hand, the 2D AFM topographies showed that the MG63 cells incubated with the Si discs and 30MgHApCh composite layers suffered changes to their morphology. round cells were observed on the surfaces of the Si discs and 30MgHApCh composite layers compared to the elongated, very well aligned MG63 cells present on the HApCh and 8MgHApCh composite layer surfaces. These findings were also confirmed by the 3D representation of the 2D AFM topographies. Moreover, the 2D AFM topographies revealed that the MG63 cells spread on the surfaces of the HApCh and 8MgHApCh and formed a monolayer with the typical characteristics of an elongated fibroblastic morphology [46,47,48,49,50,51].

**Figure 10:**
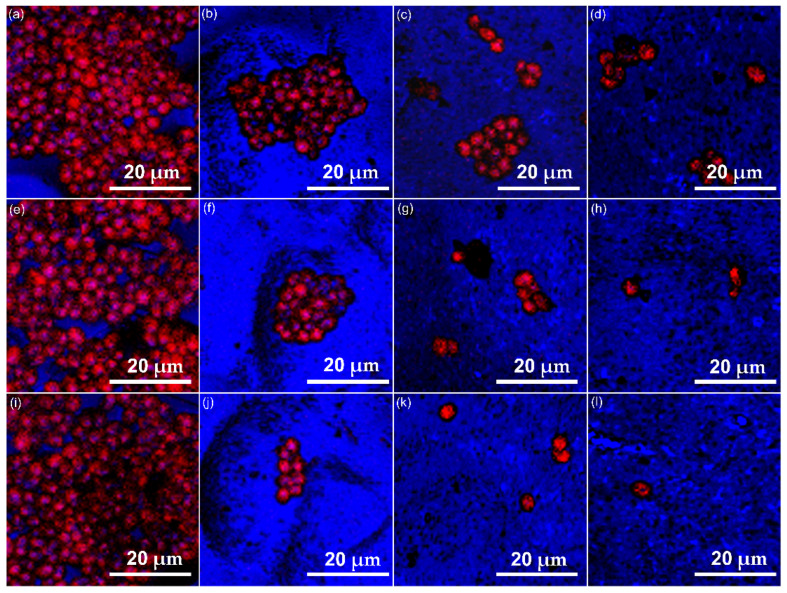
Two-dimensional CLSM images of *Candida albicans* ATCC 10231 cell development on Si discs (**a**,**e**,**i**), HApCh (**b**,**f**,**j**), 8MgHApCh (**c**,**g**,**k**) and 30MgHApCh (**d**,**h**,**l**) composite layers after 24, 48 and 72 h of incubation.

Complex information regarding the biological properties of the MgHAPCh composites layers was also obtained by the in vitro antifungal assays. The adherence and development of *C. albicans* fungal cells was assessed for three different contact time intervals (24, 48 and 72 h). The antifungal activity of the samples was assessed qualitatively by CLSM investigation. A Si disc was also used as a control for the in vitro antifungal assays. The results of the CLSM qualitative assays are depicted in Figure 10a–l. The CLSM observation emphasized that the fungal cells attached to the MgHApCh composite layers’ surfaces had the typical morphology features of *C. albicans* fungal cells with an oval shape and sizes from 2.15 to 4.74 µm. The CLSM studies showed that all the investigated samples, except for the Si discs, have successfully inhibited the development and adherence of the *C. albicans* fungal cells on their surface.

More than that, the CLSM results suggested that the control Si discs promoted the adhesion and biofilm development of fungal cells on their surface and that they had a positive influence on their proliferation. Furthermore, the CLSM data suggested that both the contact time and the tested sample had an impact on fungal cell adherence and inhibition. Furthermore, CLSM visualization emphasized that all the MgHApCh composite layers started to be effective in inhibiting the C. *albicans* fungal cell adhesion beginning with the first 24 h of contact. The CLSM images highlighted that the fungal biofilms formed on the surfaces of MgHApCh composite layers after 48 h of contact time were not organized, with the fungal cells being either isolated or forming small colonies unevenly distributed on their surface. The results depicted in Figure 10a–l demonstrated that there was a strong correlation between the contact time and the investigated sample. An increase in the antifungal activity was observed with the increase in the contact time for all the tested samples, except for the control Si discs. An increase in antifungal activity was also observed in the samples with a higher magnesium concentration. The images also showed that after 72 h, the fungal cells that adhered to the 8MgHApCh and 30MgHApCh composite layers’ surface were almost extinct.

**Figure 11:**
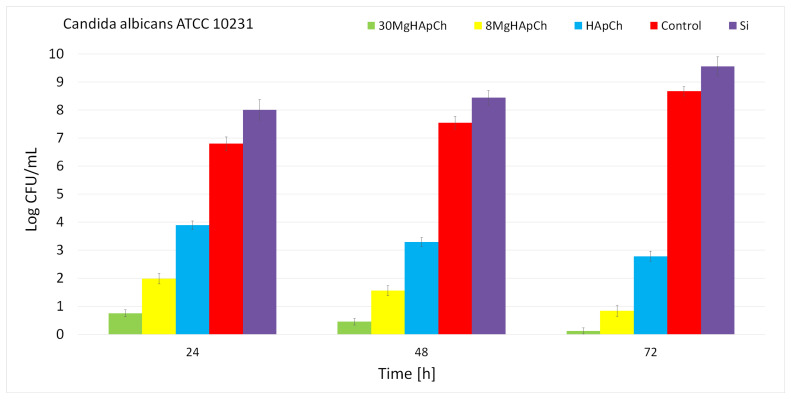
The antifungal properties of Si discs, HApCh, 8MgHApCh and 30MgHApCh composite layers against *C. albicans* (ATCC^®^ 10231) fungal cells after 24, 48 and 72 h of incubation.

The antifungal activity of the MgHApCh composite layers was also studied by performing a quantitative evaluation of the antifungal activity of the MgHApCh composite layers against *C. albicans* ATCC 10231 fungal strain. The results of the in vitro antifungal quantitative assay are presented in Figure 11. The data suggested that the fungal cells’ development was inhibited in the early 24 h of exposure time in the case of the HApCh, 8MgHApCh and 30MgHApCh composite layers compared to the control (C+). More than that, the results also demonstrated that the Si discs favored the development of *C. albicans* fungal cells compared to the fungal control. Furthermore, the results highlighted that the antifungal activity of the investigated samples was determined by both the exposure time and type of composite layers. The antifungal activity was more pronounced in the case of 8MgHApCh and 30MgHApCh and there was a strong correlation between the magnesium concentration and the antifungal activity. The strongest antifungal activity was noticed for the 30MgHApCh composite layers. These results are in good agreement with previously reported data and with the qualitative visualization by CLSM [8,22,32,33,52,53,54].

The obtained results highlighted that the MgHApCh composite layers could be taken into consideration for the development of future biocompatible devices with antifungal properties. Previous studies have reported that the biological properties of various materials and coatings could be correlated with the different intrinsic parameters of the samples and also with the biological environment they came into contact with [8,22,54,55,56]. More than that, the biological behavior of these composite layers could also be attributed to the synergistic interaction between the substrate and the composite material as well as to the physico-chemical properties of the coating material and its individual components.

On the other hand, the results of our previous studies conducted on un-irradiated chitosan-coated magnesium-doped hydroxyapatite with x_Mg_ = 0.025 obtained by the vacuum deposition method showed that the coatings are uniform and homogeneous [32]. The results of the MTT assay exhibited that the studied composite coatings possessed a good biocompatibility against the fibroblast cell line after 24 h of incubation [32]. In addition, similar results regarding the physico-chemical characteristics of magnesium-doped hydroxyapatite/chitosan coatings obtained by rf magnetron sputtering discharge have been reported by B. Bita and collaborators [57]. In the study conducted by D. Predoi and coworkers [31] the strong antifungal activity of chitosan-coated magnesium-doped hydroxyapatite (x_Mg_ = 0.1) layers obtained by spin-coating was proved against *C. albicans* fungal strain. At the same time, previous studies have demonstrated that the surface morphology and optical properties of chitosan-hydroxyapatite coatings obtained by rf magnetron sputtering discharge are influenced by the Ar gas working pressure [33]. In addition, valuable information about the influence of the Mg concentration on the biological and physico-chemical properties of the magnesium-doped hydroxyapatite in a chitosan matrix (x_Mg_ = 0, 0.08 and 0.3) coatings was reported for the first time in this study. Therefore, we could state that the results presented in this paper are in good agreement with the previous studies conducted on similar coatings.

Nonetheless, this information is scarce and there is still a need for future studies to better understand the complex interaction between engineered materials or coatings and biological tissues.

## 4. Conclusions

The main objective of this study was to obtain (by spin coating) and to investigate both the biological and physico-chemical properties of magnesium-doped hydroxyapatite in a chitosan matrix (Ca_10−x_Mg_x_(PO_4_)_6_(OH)_2_; x_Mg_ = 0, 0.08 and 0.3; HApCh, 8MgHApCh and 30MgHApCh) composite coatings. The results of the XPS and EDX studies revealed the composite coatings purities. In addition, it was noticed that the surfaces of the coatings were smooth and free of cracks. In addition, the increase in Mg concentration in the samples lead to a more structured surface. In the obtained FTIR spectra, the presence of both hydroxyapatite and chitosan vibrational peaks were observed. The cytocompatibility of the HApCh, 8MgHApCh and 30MgHApCh coatings was demonstrated on a human osteoblast-like cell line (MG63). In addition, a good antifungal activity of the coatings against *C. albicans* ATCC 10231 cells was observed. Therefore, our results suggest that these composite layers could have promising potential for developing novel biomaterials with applications in the biomedical field.

## Figures and Tables

**Table 1:** IR wavenumber (cm^−1^) assignments of HApCh sample [32,33].

Wavenumber (cm^−1^)	Assignments
963	v_1_ (PO_4_^3−^)
1095, 1029	v_3_ (PO_4_^3−^)
481	v_2_ (PO_4_^3−^)
563, 603, 635	v_4_ (PO_4_^3−^)
3332	v (OH^−^)
1465	v (C–H)
1524	v (NH_2_)

**Table 2:** Concentration of Ca, P, O and Mg (%).

HApCh		30MgHApCh	
Element	Atomic %	Element	Atomic %
Ca	34.85	Ca	32.84
P	20.87	P	21.40
O	44.28	O	42.86
Mg	0	Mg	2.9

## Data Availability

Not applicable.
